# Reinventing the Honey Industry: Opportunities of the Stingless Bee

**DOI:** 10.21315/mjms2018.25.4.1

**Published:** 2018-08-30

**Authors:** Mohd Zulkifli Mustafa, Nik Soriani Yaacob, Siti Amrah Sulaiman

**Affiliations:** 1 Department of Neurosciences, School of Medical Sciences, Health Campus, Universiti Sains Malaysia, 16150 Kubang Kerian, Kelantan, Malaysia; 2 Department of Chemical Pathology, Universiti Sains Malaysia, Health Campus, 16150 Kubang Kerian, Kelantan, Malaysia; 3 Integrative Medicine Unit, School of Medical Sciences, Universiti Sains Malaysia, Health Campus, 16150 Kubang Kerian Kelantan, Malaysia; 4 Hospital USM, Health Campus, USM, 16150, Kubang Kerian, Kelantan, Malaysia

**Keywords:** stingless bee, honey, socio-economy, complementary medicine

## Abstract

Honey is uniquely produced by honeybees (*Apis* sp.) and stingless bees (*Meliponini* sp.) and exhibits tremendous medicinal properties such as antimicrobial, anticarcinogen and antioxidant. However, it has not been included as a mainstream approach to disease management and has been disregarded in the modern pharmaceutical era. The stingless bee, which is known locally as *lebah kelulut* in Malaysia, is an important species that is well adapted for tropical countries and has emerged as an alternative source of honey. The reinventing honey quality (RHQ) project was introduced in 2012 to empower growth in the stingless bee industry, which has a direct impact on the production of high-quality honey. The objectives of the project include transforming the industry into a sustainable source of income for beekeepers, while simultaneously catalysing bee conservation activities for plant and crop pollination, thus becoming a new medium for targeting socio-economies and ecology.

Honey is uniquely produced by honeybees (*Apis* sp.) and stingless bees (*Meliponini* sp.) and exhibits tremendous medicinal properties such as anti-microbial, anti-carcinogen and antioxidant. It is specially highlighted as a natural remedy in the Holy Quran, as implied in *Surah An-Nahl* (‘the Bees’) in chapter 16, verse 69. Despite the medicinal properties, honey has never been included within the mainstream approach to disease management or classified under complementary medicine (1). Bees also act as plant pollinators, indirectly resulting in improved plant pollination and increased crop production by up to 40% (2), thus potentially providing additional income to beekeepers and their neighbourhoods, while increasing national agriculture products.

In Malaysia, production of honey by honeybees, such as *Apis melifera*, has not been profoundly successful due to the *Varroa dectructor* mite outbreak of 1996 (1). Thus, availability of local honey is completely dependent on honey hunters who obtain feral honey from stinger honeybees, such as *Tualang* bees (*Apis dorsata*). The *Tualang* bees, which mainly nest in the jungle and far off the ground, limit the implementation of standard production procedures. Meanwhile, stingless bees (*Meliponini* sp.) or *lebah kelulut* which do not have stingers, build nests in already existing cavities or hollowed out areas of trees, buildings and hives. This nesting behaviour provides the opportunity for stingless bees to be cultivated in intensive farms with controlled environmental conditions or in homes in rural areas that implement standard operating procedures. Thus, empowerment of today’s stingless bee industry would have direct impact on production of high-quality honey, while also sustaining pollination of crops and other plants, particularly to maintain biodiversity. The stingless bee is well adapted to tropical and subtropical environments, such as Malaysia, Brazil, Mexico, Africa, Northern Australia and the Southeast Asian regions. Therefore, reinventing stingless bee honey industry in tropical countries could be a new medium for targeting socio-economies and ecology.

More than 38 stingless bee species have been identified in Malaysia but only four species are commercially cultivated: *Geniotrigona thoracica, Heterotrigona itama, Lepidotrigona terminata* and *Tetragonula leviceps*. The bees produce an average of 4 kg honey per colony each year. The major composition of stingless bee honey includes sugars (fructose and glucose) with nearly zero hydroxymethylfurfural (HMF). It also contains small amounts of other compounds, such as organic acids, phenolic compounds (eg., phenolic acids and flavonoids), proteins, amino acids (eg., phenylalanine, alanine, tyrosine, valine, acetate and trigonelline), enzymes, vitamins and minerals (3). The polyphenolic content is nearly tenfold higher compared to other types of honey. The honey has great potential for prevention of chronic diseases, such as cancer, stroke, hypertension and diabetes, as measured by its ability to manipulate signalling pathway of disease development (4). Recently we showed that supplementation with honey from the stingless bee *Heterotrigona itama* led to enhancement in memory and learning in mice and increased anti-obesity parameters in high fat diet-induced obese rats, indicating the potential role of honey in controlling obesity-associated problems.

Stingless beekeeping began at the Malaysian Agricultural Research and Development Institute (MARDI) in 2007 followed by a Universiti Sains Malaysia workshop for *kelulut* honey production in 2010. In 2012, the Reinventing Honey Quality (RHQ) project (led by the corresponding author) has brought the stingless beekeeping field to light, transforming the stingless bee honey industry into a sustainable source of income for Malaysian beekeepers via premium honey production, while simultaneously catalysing bee conservation activities, thus ensuring sustainability of bees for plant pollination. The RHQ project, equipped with the artificial Mustafa-Hive (Meliponiculture Using Split-able Throne within Air-jacketed palace For Amplification-Hive), facilitates colony expansion and health screening, while simultaneously providing a habitat that can be adapted to changing climatic conditions. The Mustafa-Hive effectively promotes a more hygienic honey production practice via in-door harvest approach from systematically organised farms (Figure 1). The system had survived the first ever black soldier fly larvae infection outbreak in 2016 that caused the loss of over RM1 million. The outbreak that was associated with the El-Nino affected the colonies primarily in traditional log-hives, but no cases were reported in the Mustafa-Hive rearing system.

Malaysia’s tropical climate, coupled with its high humidity level and temperatures averaging between 28 °C–30 °C, results in honey with high moisture content, causing fermentation that produces a sour taste and foamy texture, which depreciates the value of Malaysian honey internationally. In response, the Honey Interlinked Dehydration and Dispenser Apparatus (HILDA) was invented under the RHQ project. Dehydrating the honey from 35% to 18% not only prevents fermentation but also raises both the quality and value of the final product. HILDA’s unique design represents an integrated platform for hygienic honey harvesting, water dehydration without a heating element and bottling.

The stingless bee industry above has resulted in several spill-over impacts (Figure 2), such as gardening activities that enhance nectar supplies to the bees and healthy lifestyles. Additionally, the house compound is kept clean, which subsequently curbs dengue cases and limits fogging activities that are also toxic to bees. Several downstream products of honey and propolis have helped promote the beekeepers as entrepreneurs and provided additional income to the community. A survey indicated that 36% and 29% of stingless beekeepers generate additional income of RM833 and RM1,666 monthly, respectively. Hence, this industry has contributed to holistic innovations that are beneficial socio-economically and ecologically in Malaysian perspective (5).

Productive yields of stingless bee honey are expected to influence the supply and demand of the honey industry and becoming a new commodity for the country. Stingless beekeeping transformation programmes should increase investment towards strengthening honey product chains from beekeepers that range from small, traditional activities that are labour-intensive to huge, capital-intensive industrial processes. Hive identification and permit must be registered, and the honey source should be traceable. This will possibly involve initiation of a mobile-HILDA in order to reach the rural areas to ensure small-scale beekeepers’ participation in the transformation programme. Establishment of a national institute that centralises the transformation programme of stingless beekeeping is needed, with participation from all relevant communities, organisations, academia and governments. Planning should also be directed towards specific rearing guidelines, farm and honey certifications as well as upgrading marketing aspects, especially the facilitation phase, involving export requirement specifications. National positioning of *kelulut* honey using neuromarketing tools is now important in rebranding traditional stingless bee honey at international market. Introduction of MS2683 and MS2679 2018 certification for a Malaysian standard of stingless beekeeping and honey content are among the big achievements in this field.

To support colony breeding, a genetic repository of stingless bee species should be assembled to preserve the genetic identification features of local stingless bees as well as for authentication purposes and queen bee production. Identification of local and unique *kelulut* factors and entomology signatures in honey are also crucial to improve the grade of stingless bee honey. Analysis of contents and efficacy test on honey should consider sampling of honey produced in an area of land with favourable tree species, such as coconut (*Cocos nucifera*), acacia (*Acacia mangium*), rubber (*Hevea brasiliensis*), durian (*Durio zibethinus*) and *air mata pengantin* (*Antigonon leptopus*) in the localities. Areas that are surrounded by these types of trees have shown notable achievements in honey yields, which can be introduced as plants of choice for ‘beescaping’ in the future.

Thus, upholding the stingless bee honey industry among communities serves as a holistic approach for a sustainable source of honey that can also benefit socio-economies, species survival and long-term ecological preservation.

## Figures and Tables

**Figure 1:**
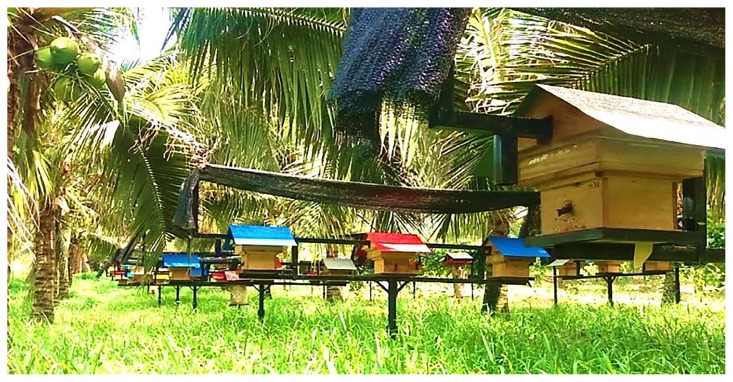
Mustafa-Hive farm that provides a well-structured, intensive farm with systematic locking that protects the hives from predators, bee enemies, monkeys, wild boars and thieves

**Figure 2:**
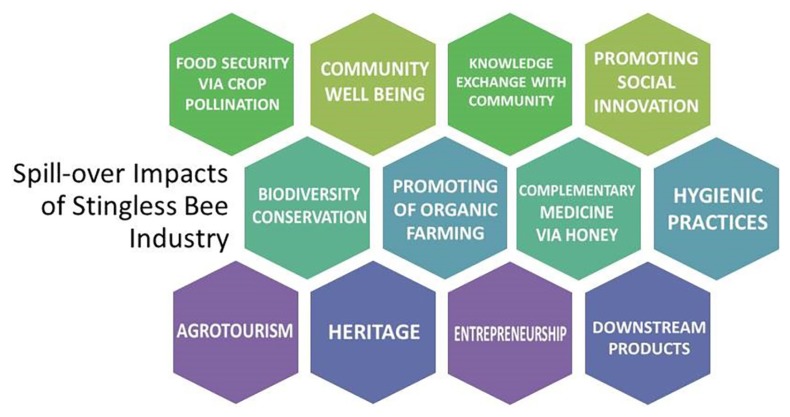
Summary of spill over impact of stingless bee industry
